# The composition of T cell infiltrates varies in primary invasive breast cancer of different molecular subtypes as well as according to tumor size and nodal status

**DOI:** 10.1007/s00428-019-02568-y

**Published:** 2019-04-17

**Authors:** Anna Glajcar, Joanna Szpor, Diana Hodorowicz-Zaniewska, Katarzyna Ewa Tyrak, Krzysztof Okoń

**Affiliations:** 1 Department of Pathomorphology, Jagiellonian University Medical College, ul. Grzegórzecka 16, 31-531 Kraków, Poland; 2 Department of General, Oncological, and Gastrointestinal Surgery, Jagiellonian University Medical College, Kraków, Poland; 3 Department of Internal Medicine, Jagiellonian University Medical College, Kraków, Poland

**Keywords:** Breast cancer, Microenvironment, Cytotoxic T cells, Regulatory T cells, Th2 cells, Tumor-infiltrating lymphocytes

## Abstract

T lymphocytes are the most numerous immune cells in tumor-associated infiltrates and include several subpopulations of either anticancer or pro-tumorigenic functions. However, the associations between levels of different T cell subsets and breast cancer molecular subtypes as well as other prognostic factors have not been fully established yet. We performed immunohistochemistry for CD8 (cytotoxic T cells (CTL)), FOXP3 (regulatory T cells (Tregs)), and GATA3 (Th2 cells) in 106 formalin-fixed paraffin-embedded invasive breast cancer tissue samples and analyzed both the numbers and percentages of investigated cells in tumor-associated infiltrates. We observed that triple-negative breast cancer (TNBC) and HER2+ non-luminal breast tumors were associated with more numerous CTLs and Tregs and a higher Treg/Th2 cell ratio as compared with luminal A subtype. A higher Treg percentage was related to a decreased hormone receptor expression, an increase in the Ki67 level, a greater tumor size of luminal tumors, and the presence of lymph node metastases. Moreover, differences in the composition of T cell infiltrates were associated with HER2 status and histologic grade and type, and a distinct immune pattern was observed in tumors of different phenotypes regarding pT stage and nodal status. The results of our work show the diversity of T cell infiltrates in primary invasive breast cancers of different phenotypes and suggest that progression of luminal or non-luminal tumors is related to distinct tumor-associated T cell composition.

## Introduction

In tumor microenvironment, lymphocytes predominate in mononuclear infiltrates and represent an adaptive antitumor immune response. The most abundant population of tumor-associated lymphocytes is T cells, which include many subpopulations differing in their function. Among them, CD8+ cytotoxic T lymphocytes (CTLs) are particularly known for their cytolytic activity against cancer cells. On the contrary, GATA3+ T helper 2 (Th2) cells and FOXP3+ regulatory T lymphocytes (Tregs) downregulate antitumor immune response by impairing antigen presentation, activity, and cytotoxicity of other immune cells, thus promoting tumor growth and immune tolerance. Tregs originate from naïve T cells both in the thymus and at the periphery, and the process of their differentiation is orchestrated by a specific cytokine milieu. Molecules secreted by tumor-infiltrating lymphocytes (TILs), cancer cells, and other components of tumor microenvironment affect the composition and function of the cancer milieu, and, thereby, modulate the course of breast cancer progression [1–6]. It was observed that TILs rich in Tregs interact with cancer-associated fibroblasts, contributing to stromal remodeling, that presumably promote tumor growth and invasion [7]. The density of T cells was reported to increase as mammary tumor progresses from normal breast tissue, through benign and in situ lesions, to invasive ductal cancers; this finding was interpreted as a stepwise increase in immunity with the course of mammary tumorigenesis [5–8]. An antitumoral immunity shows plasticity (immunoediting) and changes in time from tumor elimination (based highly on CTLs), through the equilibrium phase to immune escape (characterized by immunosuppressive profile of tumor microenvironment and generation of Tregs). The process of immunoediting is thought to result from the shifted balance between respective T cell phenotypes and selection of non-immunogenic clones [9, 10]. In line with these, the immune response appears to be dysfunctional and skewed toward suppression in invasive breast tumor tissue [2, 11].

Since the discovery of the intrinsic molecular subtypes that differ in their genetic pattern and clinical aggressiveness, invasive breast cancer has become regarded as a heterogeneous disease [12, 13]. To a certain extent, the interplay between malignant breast tumor and TILs is dependent on tumor genetics and biology [10]. Moreover, there is growing evidence that prognostic and predictive relevance of TILs varies in breast cancer of different intrinsic subtypes [1, 4, 5, 10, 11]. Nonetheless, the relationships between composition of lymphocytic milieus and breast cancer molecular subtypes have not been fully elucidated so far. Relationships between cancer and its microenvironment are of great interest, as some chemotherapeutic agents may elicit or enhance antitumor immune reactions, and innate, adaptive, cellular, and humoral pathways may be involved in cancer cell killing [14]. Simultaneously, new therapeutic approaches that aim at inducing potent immune response are sought. This includes an increase of tumor immunogenicity, inhibition of immune evasion [10, 11], and enhancement of cytotoxic and Th1 response, as well as a reduction of regulatory and Th2 cell impact on neoplastic breast tissue [5].

In our study, we investigated the lymphocyte infiltrate composition in order to assess its relationships with invasive breast cancer molecular subtypes and the occurrence of other prognostic and predictive markers for this disease. For this purpose, we evaluated both numbers of CTLs, Tregs, and Th2 cells and their percentages in tumor-associated immune infiltrates. Moreover, we also calculated proportions of investigated cells to assess differences in their relative quantities with regard to clinico-pathological indicators in breast cancer.

## Material and methods

### Material

The material comprised 106 routinely processed, formalin-fixed paraffin-embedded tissues of primary invasive breast carcinomas diagnosed between 2002 and 2015. The patients who received presurgical chemotherapy were excluded from the study. The archival hematoxylin-eosin–stained slides were re-evaluated and representative, well-preserved specimens were chosen for immunohistochemistry. The Nottingham Histologic Grade system was used for grading, and the 8th edition of the AJCC system was used for staging [15].

### Immunohistochemistry

Immunohistochemistry (IHC) for CD8, FOXP3, GATA3, estrogen receptor (ER), progesterone receptor (PR), and Ki67 protein was performed according to the protocol routinely used in our laboratory. The selected blocks were cut into 4-μm-thick sections. Antigen retrieval was performed by incubating the slides in a citrate buffer (pH 6.0; 0.01 M) or EDTA (pH 8.0; 0.01 M) at 97 °C in a water bath for 40 and 30 min, respectively. The UltraVision Quanto Detection system (Lab Vision, Thermo Fisher Scientific, USA) and 3,3′-diaminobenzidine as chromogen were used, and the slides were counterstained with Mayer hematoxylin (Thermo Fisher Scientific, Waltham, USA) and coverslipped. Immunohistochemistry for HER2 (PATHWAY 4B5, Ventana Medical Systems Inc., USA) was performed on a BenchMark BMK Classic autostainer (Ventana, USA) using an UltraView DAB Detection Kit (Ventana Medical Systems Inc., USA). The primary antibodies used are listed in Table 1.

**Table 1:** Antibodies used in the study

	Clone	Dilution	Antigen retrieval	Incubation time	Manufacturer
CD8	C8/144B	1:100	Citrate	60 min	Dako, USA
FOXP3	236A/E7	1:100	EDTA	30 min	Abcam, UK
GATA3	L50–823	1:100	EDTA	30 min	Cell Marque, USA
ER	6F11	1:100	Citrate	30 min	Novocastra (Leica Biosystems, Germany)
PR	PgR636	1:100	Citrate	60 min	Dako, USA
Ki67	MIB-1	1:100	Citrate	30 min	Dako, USA

For specimens with HER2 status 2+ in immunohistochemistry, fluorescence in situ hybridization (FISH) was conducted. FISH was performed using a PathVysion HER-2 DNA Probe Kit II (Abbott Molecular, USA) according to the manufacturer’s protocol. The red Locus Specific Identifier (LSI) HER-2/neu and green Centromere Enumeration Probe (CEP 17) signals were counted on a fluorescence microscope equipped with specific filter sets and HER-2/neu to CEP17 ratio > 2.0 was considered as HER2/neu amplification [16].

### Evaluation of immunostaining and lymphocytic infiltrates

**Fig. 1:**
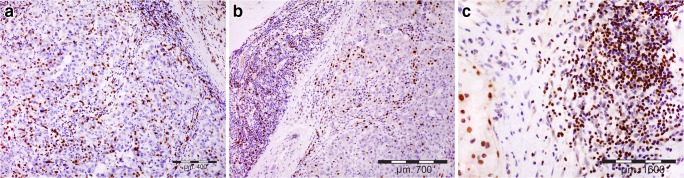
Representative infiltration of T cell subpopulations in investigated invasive breast cancer tissues; **a** CTLs, **b** Tregs, **c** Th2 cell infiltrates (at invasive margin); immunostaining for CD8, FOXP3, and GATA3, respectively; light microscopy, magnification used: × 200 (**a**, **b**) and × 400 (**c**)

The immunostained slides were initially scanned on a Nikon Labophot-2 optical microscope (Tokyo, Japan) at low magnification (× 100), and the areas with the highest number of positive cells were chosen. Then, for CD8+ and FOXP3+ T cell populations, positively stained cells were counted in 5 high-power fields (HPFs; × 400, 0.2-mm^2^ field area) and added together, which represented cell counts in 1 mm^2^ of the examined tissue. The positive cells located in tumor-surrounding stroma, no further than 1 HPF from the tumor edge, were regarded as invasive margin or tumor edge, while positive cells located within neoplastic tissue (i.e., in contact with cancer cells) were considered intratumoral or intraepithelial population (Fig. 1). Additionally, for CD8+, FOXP3+, and GATA3+ cells, the percentages of positively stained cells were visually evaluated in mononuclear infiltrate at the invasion front. The percentages of investigated cells were evaluated in 5 HPFs and averaged. Finally, the ratios of examined T cell populations were calculated separately for their numbers in the intratumoral area and at the tumor edge, as well as for their percentages in tumor-surrounding stroma. In the study, CD8+, FOXP3+, and GATA3+ were considered CTLs, Tregs, and Th2 cells, respectively.

Additionally, evaluation of TILs was performed in tumor stroma, in the whole tissue section, according to the recommendations of the International TILs Working Group 2014 [17].

Positive ER and PR expression thresholds were set when ≥ 1% of neoplastic cells showed positive immunostaining. The threshold for discriminating between low and high Ki67 expression was set at ≥ 20% of positive cells. Scoring of the HER2 staining was performed by the standard method [16].

### Definition of breast cancer molecular subtypes

The cases were classified into molecular subtypes according to the St Gallen 2015 International Expert Consensus [13]: luminal A (ER+ and PR ≥ 20%, Ki67 < 20%, HER2−), luminal B/HER2− (ER+, HER2− with PR <20% and/or Ki67 ≥ 20%), luminal B/HER2+ (ER+ or PR+, HER2+), HER2+ non-luminal (ER−/PR−/HER2+), and triple-negative breast cancer (ER−/PR−/HER2−).

### Statistical analysis

To assess the differences between groups, the ANOVA Kruskal–Wallis and Mann–Whitney *U* tests were performed. A *t* test was applied for normally distributed variables. The correlations between groups were evaluated by using the Spearman rank test. All analyses were performed using Statistica 13 (StatSoft Inc., USA). In brackets, the data are expressed as mean values ± standard deviations; *p* values < 0.05 were considered statistically significant.

## Results

A detailed description of the study group is shown in Table 2.

**Table 2:** Characteristics of the study group

Characteristic	No. of cases	Percentage
Total	106	100.0
Mean patient age 55.7 (range 29–87)		
Tumor size		
pT1	56	52.8
pT2	46	43.4
pT3	3	2.8
pT4	1	0.9
Nodal involvement		
pN0	53	50.0
pN1	32	30.2
pN2	8	7.5
pN3	12	11.3
Stage of disease		
I	40	37.7
II	42	39.6
III	22	20.7
IV	2	1.9
Nottingham histologic grade		
G1	16	15.1
G2	34	32.1
G3	56	52.8
Histological type		
NOS	93	87.7
Lobular	11	10.4
Other	2	1.9
Molecular subtype		
Luminal A	33	31.1
Luminal B	14	13.2
Luminal B/HER2+	12	11.3
HER2+ non-luminal	20	18.9
Triple negative	27	25.5

### Lymphocyte infiltrate composition in different breast cancer molecular subtypes

**Fig. 2:**
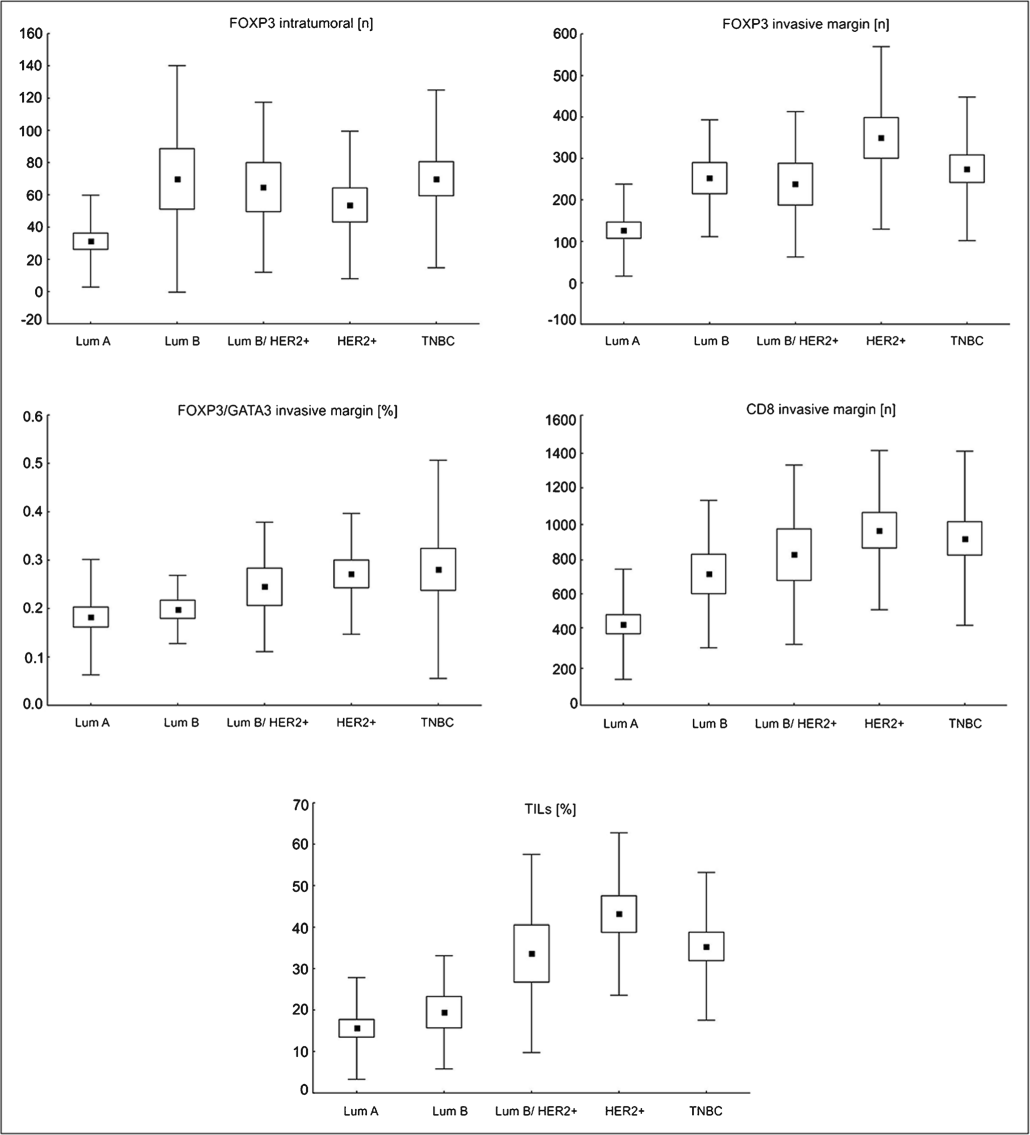
The significant differences in T cell subpopulation infiltrates of primary breast cancer tissue with reference to molecular subtypes. Lum A, luminal A; Lum B, luminal B/ HER2−; Lum B/HER2+, luminal B/HER2+; HER2+, HER2+ non-luminal; TNBC, triple-negative subtype. Central point is the arithmetic mean, box is the arithmetic mean ± standard error, and whisker is the arithmetic mean ± standard deviation.

We noted that both TNBC and HER2+ non-luminal tumors were more abundantly infiltrated by lymphocytes, as seen on H&E sections, in comparison with luminal A lesions (*p* < 0.001). Moreover, the HER2+ non-luminal subtype was also associated with a higher TIL level than lesions of luminal B phenotype (*p* < 0.007). With reference to the numbers of individual lymphocyte populations, we observed significantly more CTLs at the invasive margin of TNBC and HER2+ non-luminal cancers than in luminal A tumors (*p* < 0.001 for both comparisons). The same observation was made for Tregs of the tumor edge (TNBC vs. luminal A: *p* < 0.006; HER2+ non-luminal vs luminal A: *p* < 0.001), and this population was more abundant in luminal B cancers, as compared with luminal A tumors (*p* < 0.050). The intratumoral Treg level was also significantly higher in TNBC than in luminal A tumors (*p* < 0.003). No statistically significant differences were observed for CTL/Treg number ratio both within neoplastic epithelium and at the invasive margin (Fig. 2, Tables 3 and 4). Regarding the percentages of analyzed T cell populations in tumor-associated infiltrates, we observed higher Treg/Th2 cell percentage ratio at the tumor edge of HER2+ non-luminal as compared with luminal A lesions (*p* < 0.040; Fig. 2, Table 4).

**Table 3:** Associations between quantities of investigated T cell populations and selected prognostic factors in breast cancers

	Intratumoral									Invasive edge stroma								
	CD8 (n)			FOXP3 (n)			CD8/FOXP3			CD8 (n)			FOXP3 (n)			CD8/FOXP3		
	Mean	SD	*p*	Mean	SD	*p*	Mean	SD	*p*	Mean	SD	*p*	Mean	SD	*p*	Mean	SD	*p*
St Gallen 2015 molecular subtype																		
Luminal A	106.1	102.4	NS	31.3	28.5	< 0.005	4.62	3.52	NS	445.9	303.6	< 0.001	127.0	110.8	< 0.001	5.29	3.74	NS
Luminal B	141.1	105.1		69.9	70.3		3.79	3.36		723.2	406.4		252.2	140.9		3.89	3.28	
Luminal B/HER2+	133.4	82.1		64.7	52.7		2.81	1.57		829.5	493.6		237.7	175.3		4.39	2.30	
HER2+ non-luminal	126.5	132.6		53.7	45.7		2.88	1.98		964.4	439.1		349.4	219.9		3.39	1.89	
TNBC	234.6	257.0		70.0	55.1		3.39	2.36		919.5	480.2		275.0	173.1		3.92	1.83	
Histological grade																		
G1	62.3	34.7	< 0.002	26.6	26.1	< 0.001	2.95	1.31	NS	420.4	259.4	< 0.001	112.7	83.2	< 0.001	5.11	3.62	NS
G2	119.5	107.1		43.0	41.2		4.69	3.96		571.9	367.9		176.9	129.8		4.63	3.32	
G3	193.2	200.5		68.7	56.2		3.25	2.12		941.7	465.5		307.5	194.0		3.85	2.20	
HER2 overexpression																		
No	159.6	183.1	NS	53.0	52.1	NS	4.00	3.12	NS	671.1	445.2	< 0.010	205.7	157.1	< 0.008	4.52	3.10	NS
Yes	129.2	114.2		58.0	48.0		2.85	1.81		913.8	457.2		307.5	208.7		3.77	2.08	

Kruskal–Wallis test was performed

*NS* not significant, *n* cell number counted

**Table 4:** Associations between the percentage of analyzed cells in stromal lymphocytic infiltrates at the invasive margin and selected prognostic factors in breast cancer

	CD8 (%)			FOXP3 (%)			GATA3 (%)			CD8/FOXP3			CD8/GATA3			FOXP3/GATA3			TILs (%)		
	Mean	SD	*p*	Mean	SD	*p*	Mean	SD	*p*	Mean	SD	*p*	Mean	SD	*p*	Mean	SD	*p*	Mean	SD	*p*
St Gallen 2015 molecular subtype																					
Luminal A	33.2	11.8	NS	9.1	4.5	NS	52.5	11.5	NS	4.86	3.80	NS	0.64	0.21	NS	0.18	0.12	< 0.025	15.6	12.2	< 0.001
Luminal B	35.9	11.5		10.4	1.8		55.4	11.9		3.52	1.08		0.65	0.18		0.20	0.07		19.5	13.6	
Luminal B/HER2+	43.8	26.6		11.0	5.2		49.0	15.1		4.93	4.82		0.93	0.48		0.24	0.13		33.6	23.9	
HER2+ non-luminal	34.1	12.3		12.1	4.9		49.0	12.2		3.18	1.46		0.75	0.35		0.27	0.12		43.1	19.6	
TNBC	32.8	11.0		10.2	2.6		45.4	16.0		3.46	1.70		0.89	0.74		0.28	0.22		35.3	17.8	
Histological grade																					
G1	30.3	12.6	NS	8.3	4.9	< 0.015	50.8	14.5	NS	5.26	4.70	NS	0.61	0.23	NS	0.19	0.14	< 0.035	14.1	12.5	< 0.001
G2	34.1	11.9		10.5	4.5		52.6	11.7		3.77	2.14		0.66	0.22		0.20	0.10		19.5	12.3	
G3	36.6	15.8		10.8	3.4		48.3	14.3		3.79	2.64		0.86	0.59		0.26	0.18		37.8	20.6	
HER2 overexpression																					
No	33.6	11.4	NS	9.7	3.4	NS	50.5	13.8	NS	4.08	2.82	NS	0.73	0.49	NS	0.22	0.16	NS	23.6	17.2	< 0.001
Yes	37.8	19.2		11.7	4.9		49.0	13.2		3.84	3.21		0.82	0.41		0.26	0.13		39.6	21.4	

Kruskal–Wallis test was performed

*NS* not significant, *%* cell percentage evaluated

According to St Gallen 2015 distinction between luminal A and B molecular subtypes, the latter was characterized by higher numbers of CTLs (723.2 ± 406.4 vs. 445.8 ± 303.6, *p* < 0.025) and Tregs (252.2 ± 140.9 vs. 127.0 ± 110.8, *p* < 0.005) located at the invasive margin of a tumor.

As far as the HER2 status was concerned, we found that more abundant TILs as well as more numerous CTLs and Tregs at the invasion front were associated with HER2 overexpression (*p* < 0.001, *p* < 0.010, and *p* < 0.001, respectively; Tables 3 and 4).

The expression of hormone receptors showed a negative correlation with CTL counts at the tumor edge (ER: *R* = − 0.46, PR: − 0.47, *p* < 0.001), intratumoral and invasive margin Treg numbers (ER: *R* = − 0.26, *p* < 0.008 and *R* = − 0.44, *p* < 0.001; PR: *R* = − 0.26, *p* < 0.007 and *R* = − 0.45, *p* < 0.001 respectively), the percentage of Tregs at tumor edge (ER: not significant, PR: *R* = − 0.21, *p* < 0.035), TIL level (ER: *R* = − 0.57, PR: *R* = − 0.54, *p* < 0.001), and the Treg/Th2 cell percentage ratio (ER: *R* = − 0.34, *p* < 0.002; PR: *R* = − 0.31 *p* < 0.001) as well as a positive correlation with both Th2 cell percentage (ER: *R* = 0.25, *p* < 0.015; PR: *R* = 0.19, *p* < 0.050) and CTL/Th2 cell percentage ratio (ER: *R* = 0.21, *p* < 0.035; PR: *R* = 0.22, *p* < 0.030) at tumor edge. Expression of Ki67 in neoplastic cells correlated positively with CTL and Treg counts both within tumor and at the invasive margin (CTLs: *R* = 0.28, *p* < 0.005 and *R* = 0.41, *p* < 0.001; Tregs: *R* = 0.42 and *R* = 0.38, *p* < 0.001, respectively), Treg percentage at invasion front (*R* = 0.27, *p* < 0.007), TIL infiltrate (*R* = 0.46, *p* < 0.001), and Treg/Th2 cell percentage ratio (*R* = 0.29, *p* < 0.004).

### The relationships between lymphocyte infiltrate composition and other prognostic indicators in breast cancer

With regard to the tumor size, we stratified the analyzed samples into small tumors (pT1) and the lesions of diameter greater than 2 cm (pT > 1). We observed that the latter was characterized by more abundant TIL infiltrates (33.6 ± 20.7 vs. 23.8 ± 18.2, *p* < 0.007), higher intratumoral CTL number (172.7 ± 146.8 vs. 131.3 ± 179.9, *p* < 0.007), and higher Treg/Th2 cell percentage ratio at the invasion front (0.25 ± 0.12 vs. 0.22 ± 0.18, *p* < 0.045). When cancers of different phenotypes were analyzed separately, in cases of luminal cancers, a greater tumor diameter was associated with more numerous intratumoral CTLs (90.2 ± 67.0 vs. 170.1 ± 123.0, *p* < 0.006) and Tregs (35.9 ± 39.8 vs. 66.5 ± 57.9, *p* < 0.010), higher Treg percentage at the tumor edge (8.9 ± 4.1 vs. 11.2 ± 4.1, *p* < 0.020), TIL level (16.4 ± 14.8 vs. 26.4 ± 18.4, *p* < 0.025), and Treg/Th2 cell percentage ratio at invasive margin (0.18 ± 0.12 vs. 0.23 ± 0.09, *p* < 0.015). No significant differences were observed between the composition of lymphocytic infiltrate and tumor size in the non-luminal group.

**Fig. 3:**
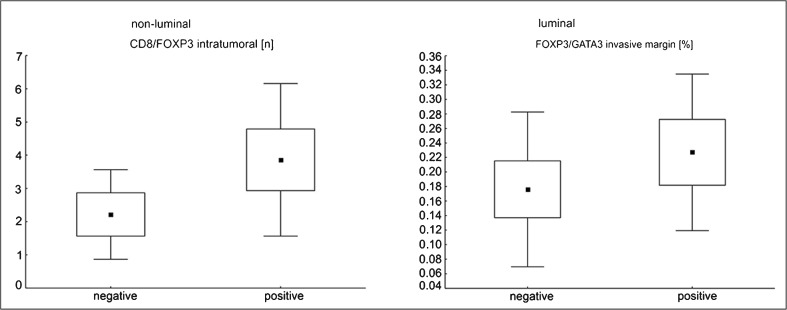
The significant differences in T cell subpopulation infiltrate of primary breast cancer tissue with reference to nodal status, after stratification upon breast cancer phenotype. Central point is the arithmetic mean, box is the arithmetic mean ± 2*standard error, and whisker is the arithmetic mean ± 0.95*standard deviation.

Primary breast cancers that developed nodal metastases showed more prominent TILs (32.9 ± 19.3 vs. 24.6 ± 19.7, *p* < 0.015), higher Treg percentage (11.2 ± 4.2 vs. 9.4 ± 3.7, *p* < 0.020), and Treg/Th2 cell percentage ratio at the invasion front (0.25 ± 0.13 vs. 0.21 ± 0.18, *p* < 0.030) in comparison with metastasis-free cases. After stratification into luminal and non-luminal cancers, we noted that a higher intratumoral CTL/Treg number ratio was associated with regional lymph node metastases (*p* < 0.035) in the latter group. For luminal tumors, there was a tendency toward higher Treg/Th2 cell percentage ratio at the edge of tumors of positive nodal status (*p* = 0.051; Fig. 3).

In terms of tumor histologic grade, we observed that tumor-associated infiltrates differed in numbers of CTLs in intratumoral area and tumor edge (*p* < 0.002 and *p* < 0.001, respectively) as well as in both populations of Tregs (intratumoral *p* < 0.003, invasive margin *p* < 0.001) and TIL abundance (*p* < 0.001) as well as in the percentage of Treg lymphocytes (*p* < 0.008) and the Treg/Th2 cell percentage ratio at the invasion front (*p* < 0.035), with their increased levels in G3 as compared to G1 tumors. Additionally, for Treg numbers, both in intratumoral and invasive margin location, CTL numbers at the tumor edge, and TIL infiltrates, significantly higher levels were noted in G3 than in G2 cancers (*p* < 0.040, *p* < 0.006, *p* < 0.001, and *p* < 0.001, respectively; Tables 3 and 4).

As far as the histologic type was concerned, NOS cancers were characterized by more abundant TILs (30.3 ± 20.3 vs. 13.1 ± 9.3, *p* < 0.003), more numerous CTLs of invasive margin (793.1 ± 461.7 vs. 380.0 ± 286.1, *p* < 0.003) and Tregs at the tumor edge (256.4 ± 180.8 vs. 101.0 ± 96.2, *p* < 0.002), and a higher Treg percentage at invasion front (10.6 ± 4.1 vs. 8.6 ± 3.9, *p* < 0.050) as well as decreased intraepithelial CTL/Treg number ratio (3.27 ± 2.43 vs. 7.09 ± 3.86, *p* < 0.001) as compared with CLI lesions.

The tumor tissue excisions obtained from stage I patients were related to a significantly lower TIL level (19.7 ± 15.1 vs. 36.1 ± 22.5, *p* < 0.001), lower counts of CTLs (596.8 ± 449.8 vs. 864.3 ± 472.1, *p* < 0.015), and Tregs at invasive margin (176.3 ± 152.8 vs. 288.7 ± 206.7, *p* < 0.015) as well as a lower Treg percentage (9.0 ± 4.1 vs. 10.5 ± 2.8, *p* < 0.045) and Treg/Th2 cell percentage ratio at the tumor edge (0.20 ± 0.20 vs. 0.24 ± 0.12, *p* < 0.035) in comparison with stage II cancers; for the two latter parameters, such a difference was also observed between stage I and stage III/IV cancers (stage III/IV: Treg percentage − 12.3 ± 5.0, *p* < 0.009, Treg/Th2 cell percentage ratio − 0.26 ± 0.12, *p* < 0.020).

## Discussion

CTLs are commonly considered as a part of cancer immune surveillance. Some research into breast cancer-linked CTLs revealed their lytic and proapoptotic activity [18] as well as a memory phenotype in the majority of this cell population, particularly when high-grade lesions were concerned [19]. Tsang et al. [20] observed that CD8+ and FOXP3+ expressions were mutually exclusive in double immunohistochemical staining of breast cancer microenvironment. On the contrary, the existence of fractions of CTLs that express FOXP3 secrete immunosuppressive interleukin (IL)-10 [21] and co-express molecules associated with anergy, exhaustion, or unresponsiveness in tumor-infiltrating lymphocytes [22] was reported.

The data on associations between lymphocytic infiltrate and breast cancer molecular subtype are inconclusive. In line with our study, some authors reported that increased CTL counts were associated with ER and PR negativity, HER2 overexpression [23–25], and higher Ki67 level [25, 26] in breast cancer, while the results from other publications question these findings [20, 26, 27]. Liu et al. hypothesized that the location of immune infiltrates (intraepithelial or peritumoral) may influence activation of its cells, as cell populations within a tumor are dispersed and their interactions are impeded [23]. In the Miyan et al. study, a significantly increased number of CTLs was observed at the invasive margin of basal-like and luminal B/HER2+, with their lowest counts in luminal A lesions [25]; however, the authors applied St Gallen 2013 molecular subtype classification in their research. Tsang et al. suggested that the mechanism of intratumoral recruitment and survival of lymphocytes may differ between subtypes, and that in HER2-positive tumors, CTL migration is preferred over Treg influx [20]. Our observation of high CTL counts in HER2+ non-luminal and triple-negative phenotypes was made for a population of these cells located at tumor edge, exclusively. On the other hand, the percentages of CTLs in tumor-associated infiltrates at invasive margin did not differ between subtypes. Therefore, the increased numbers of CTLs may reflect more abundant TILs noted in these breast cancer subtypes rather than a shift in immune response toward more potent cell killing. A favorable prognostic value of high levels of breast cancer-related CTLs was attributed to ER-negative as well as ER+/HER2+ phenotypes [28]. Moreover, high FAS protein expression in ER-negative cancers was proposed to be one of the contributing factors to beneficial impact of CTL on patient survival. Of note, their adverse effect on ER-positive FAS-high patient outcome indicated different functions of tumor-infiltrating CTLs with respect to breast cancer subtypes [29].

Literature data on relationships between CTLs and other prognostic factors in invasive breast cancer are also ambiguous. Some authors [14, 19, 23–25], but not all [23, 27, 30], report higher CTL counts in tumors of higher histological grade and size, which is in line with our findings. We noted that relationships between more numerous CTL infiltrates and higher grades concerned both intraepithelial area and tumor edge, while tumors of greater diameter were characterized only by a more abundant intraepithelial population. After stratification, the latter finding remained significant for luminal lesions, exclusively. Regarding lymph node metastases, higher counts [26, 27, 31] as well as a higher frequency of CTLs [19] were observed in primary tumors with nodal spread, but such association was not observed by other groups [23, 32]. In our study, the CTL infiltrates did not differ according to nodal status.

FOXP3 is a transcription marker expressed in a vast majority of breast cancer-infiltrating Tregs [33]. Similar to CTLs, Tregs were observed to accumulate in breast tumor and its immediate milieu, in comparison with normal tissue. Moreover, tumor-infiltrating Tregs were more frequently characterized by activated, strongly immunosuppressive but exhausted phenotype, which may correspond with immune tolerance [22]. As far as the breast cancer intrinsic subtypes were concerned, the most numerous regulatory T cell infiltrate is frequently associated with either TNBC or HER2+ non-luminal phenotype of tumors, while the lowest Treg numbers are observed in luminal A lesions [23, 25, 34], which is in accordance with our results. Moreover, the stronger Treg infiltrate of TNBC concerns both the surrounding stroma and tumor center. Such findings indicated associations between tumor biological features and immunological response in invasive breast cancers [25] as well as more immunosuppressive microenvironment of clinically aggressive subtypes [23]. Moreover, the increased proportion of Tregs in a lymphocytic milieu [35], as well as their higher quantities, was noted in TNBC and hormone receptor (HR)-negative and HER2-overexpressed breast tumors [23, 34], which supports our results. We also observed slight correlations between Treg percentage in immune cell infiltrates of invasive edge and either a drop of PR or an increase in Ki67 expression that suggest a shift toward a more immunotolerant milieu in PR-negative or intensively proliferating breast cancers. More abundant Tregs were found in the center of ER-positive and in the peritumoral area of ER−/HER2+ cancers, while a lower number of cells infiltrated the intratumoral site of ER−/HER2+ tumors in Tsang et al. research [20]. Some authors did not observe any differences in Treg infiltrates regarding breast cancer intrinsic subtypes [36]. In ER-negative tumors, more intensive Treg infiltration was associated with better disease-free survival [33]. Similar to CTLs, higher levels of Tregs were often observed in high-grade cancers [14, 23, 35], but their relationship with tumor size and nodal status is controversial [22, 23, 26, 34]. We found increased Treg numbers and percentages in luminal breast tumors of greater size, as well as a higher proportion of Tregs in the microenvironment of node-positive invasive tumors, that suggests a regulatory bias in TILs of more advanced cancers.

The ratio of CTL to Treg numbers is regarded as an indicator of cytotoxicity. A higher CD8+/FOXP3+ ratio was observed by Liu et al. in the peritumoral area of non-luminal breast cancers and indicated greater cytolytic potential of the lymphocytic milieu surrounding these tumors [23]. It was postulated that the change of this parameter is rather due to Treg reduction than CTL recruitment [14]. Complementary FOXP3+/CD8+ cell ratio was suggested to reflect an immune evasion of a tumor, with its higher values in tumor center as compared with the peritumoral area. In breast tumor, it was associated with ER negativity, higher proliferation rate, and high histological grade, but not with tumor size or nodal involvement [25]. In our study, increased intraepithelial CTL/Treg number ratio was associated with lobular histology and metastatic disease of non-luminal cancers. Thus, we hypothesize that, for breast tumors of non-luminal phenotype, their spread is associated with cytotoxicity failure.

Information on tumor-associated Th2 cells in breast cancer is scarce. Th2 was reported as a predominant population of T helper cells in a mouse model of luminal breast cancer; their lower counts in the tumor milieu were associated with decreased pulmonary metastasis by Zhang et al. [37]. Moreover, higher levels of Th2 cytokines—IL-10 [14] and IL-5 [3]—were related to the lack of pathologic complete response after chemotherapy and worse survival in breast cancer patients, respectively. In our study, tumor-associated Th2 cells showed a slight correlation with HR. On the contrary, the expression of genes related to Th2 signaling was more prominent in basal cancers by Kristensen et al. [38]. Ghirelli et al. showed that cytokines secreted by breast cancer tissue resulted in regulatory Th2 bias of tumor-related immunity, which supposedly was GATA3 independent [39]. To assess immunoregulatory and suppressive potential of immune infiltrates, we evaluated the Treg/Th2 cell percentage ratio. Its high value was associated with adverse prognostic indicators: HER2+ non-luminal subtype, decrease in HR expression, increasing proliferation rate, and higher grade as well as greater tumor size and positive lymph node status, particularly in luminal cancers. In addition, the higher percentage ratio of CTLs to Th2 at invasion front modestly correlated with HR expression. Thus, we hypothesize that the bias of immunosuppressive microenvironment toward regulatory function is associated with clinically more aggressive breast cancers.

Our recent research studies aimed at evaluation of tumor microenvironment in primary invasive breast cancer have shown associations between higher quantities of mast cells and beneficial prognostic indicators [40] and relationships between T cell, B cell, and NK cell infiltrates and adverse clinical factors [41]. These findings induced us to presently investigate infiltrates of several T cell subpopulations in this disease. In conclusion, we observed that T cell infiltrates of primary invasive breast tumors differ in numbers and percentages of its individual populations regarding cancer molecular features and prognostic markers. Moreover, the relationships between lymphocytic composition and pT or nodal status vary according to cancer phenotype, suggesting that distinct mechanisms govern cancer progression in luminal and non-luminal lesions. As more numerous T cell subpopulations in breast cancer tissue may result from higher TIL levels, we additionally evaluated percentages of analyzed cells in tumor-surrounding infiltrates that should be resistant to the number of TILs. Thus, further investigation is needed to elucidate function of immune infiltrates in breast cancers of different molecular subtypes.
